# SNRPB promotes cell cycle progression in thyroid carcinoma via inhibiting p53

**DOI:** 10.1515/med-2022-0531

**Published:** 2022-10-18

**Authors:** Yan Deng, Xin Li, Wenlei Jiang, Jindan Tang

**Affiliations:** Department of Nuclear Medicine, Wuhan Fifth Hospital, Wuhan, 430050 Hubei, China; Department of Emergency, Wuhan Fifth Hospital, Wuhan, 430050 Hubei, China; Department of Nursing, Wuhan Fifth Hospital, No. 122, Xianzheng Street, Hanyang District, Wuhan, 430050 Hubei, China

**Keywords:** thyroid carcinoma, SNRPB, p53, cell cycle

## Abstract

Papillary thyroid carcinoma (PTC) accounts for more than 80% of all thyroid carcinoma cases. Small nuclear ribonucleoprotein polypeptides B and B1 (SNRPB) has been indicated to be carcinogenic in several cancers; however, its function and mechanism in PTC are unclarified. Real time quantitative polymerase chain reaction and western blotting revealed the upregulation of SNRPB and downregulation of tumor protein p53 in PTC tissues compared with the normal tissues. Flow cytometry and western blotting displayed that SNRPB silencing induced cell cycle arrest at G1 phase and suppressed the expression levels of Cyclin family proteins in PTC cells. *In vivo* experiments suggested that SNRPB silencing inhibited PTC tumor growth in mice. Bioinformatics analysis revealed that the expression of SNRPB and cell cycle-associated genes in thyroid carcinoma tissues is positively correlated. Immunofluorescence staining and co-immunoprecipitation demonstrated that SNRPB directly interacted with p53 and suppressed its expression in PTC cells. In conclusion, SNRPB facilitates cell cycle progression in PTC by inhibiting p53 expression.

## 1 Introduction

Thyroid carcinoma (THCA) is the most prevalent endocrine malignancy which accounts for approximately 12% of newly diagnosed cancers in adolescents [1]. THCA is more common in women; it was estimated that women accounted for over 31,900 cases of THCA out of a total of 48,000 new diagnoses in USA [1]. Papillary thyroid carcinoma (PTC) is the most common subtype of THCA, which comprises more than 80% of all THCA cases [2]. Current treatments including thyroidectomy and radioactive iodine have displayed favorable prognosis in most of PTC patients [3]. Nevertheless, in certain patients, the survival is still far from satisfactory due to tumor recurrence and metastasis [3,4]. For patients with locally advanced or distant metastatic PTC, the existing treatments are still insufficient [5]. Hence, having a better understanding of the underlying mechanism and finding novel effective approaches for PTC treatment are greatly demanded.

Small nuclear ribonucleoprotein polypeptides B and B1 (SNRPB) is a core component of the spliceosome and functions as a splicing factor [6]. SNRPB exerts a crucial effect on the alternative splicing process of pre-mRNAs [7]. It has been indicated that silencing of SNRP proteins inhibits cancer cell viability by modulating expression of their downstream genes [7]. As one of the SNRP proteins, SNRPB has been shown to act as an oncogene in several cancers. For example, SNRPB contributes to the malignant proliferation of hepatocellular carcinoma cells [8]. Additionally, SNRPB depletion suppresses cell growth in glioblastoma, indicating its carcinogenic role [9]. Importantly, bioinformatics analysis displayed that SNRPB is differentially expressed in different stages of THCA patients, suggesting that SNRPB might be implicated in the pathogenesis of THCA. Nevertheless, to our knowledge, the detailed role of SNRPB as well as its underlying mechanism in PTC is unanswered.

Tumor protein p53 encodes a tumor suppressor protein which responds to a diversity of cellular stresses such as oncogene activation, hyperoxia and DNA damage [10]. In response to various cellular stresses, p53 is activated by post-transcriptional modifications and oligomerization; activated p53 regulates transcription of downstream genes, consequently resulting in cell cycle arrest, apoptosis or DNA repair [11,12]. Thus, p53 is considered as a vital factor in preventing tumorigenesis [13]. Importantly, studies have demonstrated the involvement of p53 in PTC [14,15]. Nevertheless, the relationship between p53 and SNRPB in PTC is unclarified.

Herein, we probed the function of SNRPB as well as its underlying mechanisms in PTC. It was hypothesized that SNRPB affected the progression of PTC by modulating p53. The results might develop a new clue for treating PTC.

## 2 Materials and methods

### 2.1 Tissue specimens

Paired PTC and nontumor tissues (*N* = 33) were collected from PTC patients who underwent surgeries in Wuhan Fifth Hospital (Hubei, China). None of these patients received radiotherapy or chemotherapy before surgery and patients with other malignancies were excluded from the study. Written informed consent was obtained from all participants. Tissue samples were immediately frozen in liquid nitrogen and preserved at −80°C. The study was approved by the Ethics Committee of Wuhan Fifth Hospital (Hubei, China).

### 2.2 Cell culture and transfection

PTC cell lines (TPC-1 and IHH4) were obtained from WHELAB (Shanghai, China) and incubated in Dulbecco’s modified eagle medium (Invitrogen, Carlsbad, CA, USA) containing 10% fetal bovine serum (Gibco, Rockville, MD, USA) and 1% penicillin–streptomycin (Invitrogen) at 37°C in a humidified incubator with 5% CO_2_.

For knockdown assays, short hairpin RNAs targeting SNRPB (sh-SNRPB) and corresponding negative control obtained from Sangon (Shanghai, China) were transfected into PTC cells using Lipofectamine 2000 (Invitrogen). Mock served as a control group. After 48 h, the transfection efficiency was assessed by real time quantitative polymerase chain reaction (RT-qPCR). Sh-SNPRB#1 with the best silencing effect was used for subsequent experiments.

### 2.3 *In vivo* xenograft experiments

BALB/c nude mice (female, 5-week-old) were purchased from Vital River (Beijing, China) and divided into two groups, with five per group. TPC-1 cells stably transfected with sh-SNRPB or sh-NC were injected subcutaneously into the mice (4 × 10^5^ cells/mouse). Tumor volume was monitored every 3 days and calculated with the formula: volume = 0.5 × length × width^2^. After 27 days, mice were euthanized under anesthesia with 2% pentobarbital sodium (30 mg/kg). Tumors were harvested and weighed. All animal experiments were approved by the Animal Research Ethics Committee of Wuhan Fifth Hospital (Hubei, China).

### 2.4 RT-qPCR

Total RNA was extracted from tissues and PTC cells using TRIzol reagent (Invitrogen). Approximately 1 μg of total RNA was reverse transcribed using PrimeScript^TM^ RT Master Mix (Takara, Dalian, China) to obtain cDNA. RT-qPCR was implemented using SYBR Premix Ex Taq^TM^ (Takara) on a Bio-Rad Real-Time PCR System (Bio-Rad, Hercules, CA, USA). Relative SNRPB expression was calculated with the 2^−ΔΔCt^ method, with GAPDH as the internal control. Primer sequences are as follows:

SNRPB

Forward: 5′-GGAAGAGAAGCGAGTCCTC-3′

Reverse: 5′-AATACCAGTATCTTTGGGAGGAG-3′

GAPDH

Forward: 5′-TCAAGATCATCAGCAATGCC-3′

Reverse: 5′-CGATACCAAAGTTGTCATGGA-3′

### 2.5 Western blotting

Proteins were isolated from tissues and PTC cells using RIPA lysis buffer (Beyotime, Shanghai, China) and quantified with a BCA assay kit (Bio-Rad). Equal amounts of protein samples (20 μg) were separated by 10% SDS-PAGE and blotted on polyvinylidene fluoride membranes (Millipore, Billerica, MA, USA). After blocking with 5% defatted milk, the membranes were incubated at 4°C overnight with primary antibodies against: SNRPB (ab155026, 1:1,000), Cyclin A1 (ab270940, 1:1,000), Cyclin B1 (ab181593, 1:2,000), Cyclin D1 (ab16663, 1:200), Cyclin E1 (ab33911, 1:1,000), p53 (ab32389, 1:10,000) and GAPDH (ab9485, 1:2,500) (all from Abcam, Cambridge, MA, USA), followed by incubation with the secondary antibody (Abcam) at room temperature for 2 h. Eventually, protein bands were visualized with an enhanced chemiluminescence detection system (Pierce, Rockford, IL, USA) and quantified with ImageJ software (National Institutes of Health, Bethesda, MD, USA).

### 2.6 Flow cytometry

Cell cycle distribution was determined by flow cytometry analysis. Cells were fixed in 70% ethanol at 4°C overnight. After washing with phosphate buffer saline, cells were incubated with 10 mg/mL RNA enzyme (Sigma-Aldrich, St. Louis, MO, USA) and 0.5 μg/mL propidium iodide and maintained for 30 min at room temperature away from light. Afterwards, cell populations in different phases were measured by Flow Cytometer (Beckman Coulter, Inc., Brea, CA, USA).

### 2.7 Immunofluorescence (IF) staining

Cells were incubated on glass coverslips and fixed with 4% formaldehyde for 10 min, followed by permeation in 0.5% Triton X-100 for 15 min at room temperature [16]. The coverslips were blocked with goat serum (Sigma-Aldrich). Afterwards, cells were incubated with anti-SNRPB (Abcam) and anti-p53 (Abcam) primary antibodies at 4°C overnight, followed by incubation with the secondary antibody for 1 h at 37°C. Cell nuclei were stained with DAPI (Sigma-Aldrich). The fluorescence was observed under a LEICA TCS SP5 Confocal Microscope (Leica, Germany).

### 2.8 Co-immunoprecipitation (Co-IP) assay

Cells were lysed in Pierce IP lysis buffer (Thermo Scientific, Waltham, MA, USA). Cell lysates were incubated with the indicated antibodies at 4°C overnight on a rocker, followed by incubation with Protein A/G Agarose (Beyotime) for 3 h at 4°C. Then, the immunoprecipitates were analyzed by western blotting. Full-length sequences of p53 and SNRPB were inserted into pcDNA5-HA and pcDNA5-Flag vectors to construct pcDNA5-HA-p53 and pcDNA5-Flag-SNRPB [17,18]. PTC cells were transfected with the above vectors and then lysed in ice-cold IP buffer. Cell lysates were incubated with anti-Flag M2 Magnetic beads (Sigma-Aldrich) and Pierce anti-HA Magnetic beads (Thermo Scientific) overnight at 4°C. Then, the immune complexes were analyzed by western blotting using the indicated primary and secondary antibodies.

### 2.9 Statistical analysis

Data were analyzed by SPSS 21.0 software (SPSS Inc., Chicago, IL, USA) and are presented as the mean ± standard deviation. Student’s *t*-test was used for difference comparisons between two groups, while analysis of variance (ANOVA) was used for those among multiple groups followed by Tukey’s *post hoc* analysis. Each experiment was implemented in triplicate. *p* < 0.05 was considered as statistically significant.

## 3 Results

### 3.1 SNRPB displays a high level in PTC

**Figure 1:**
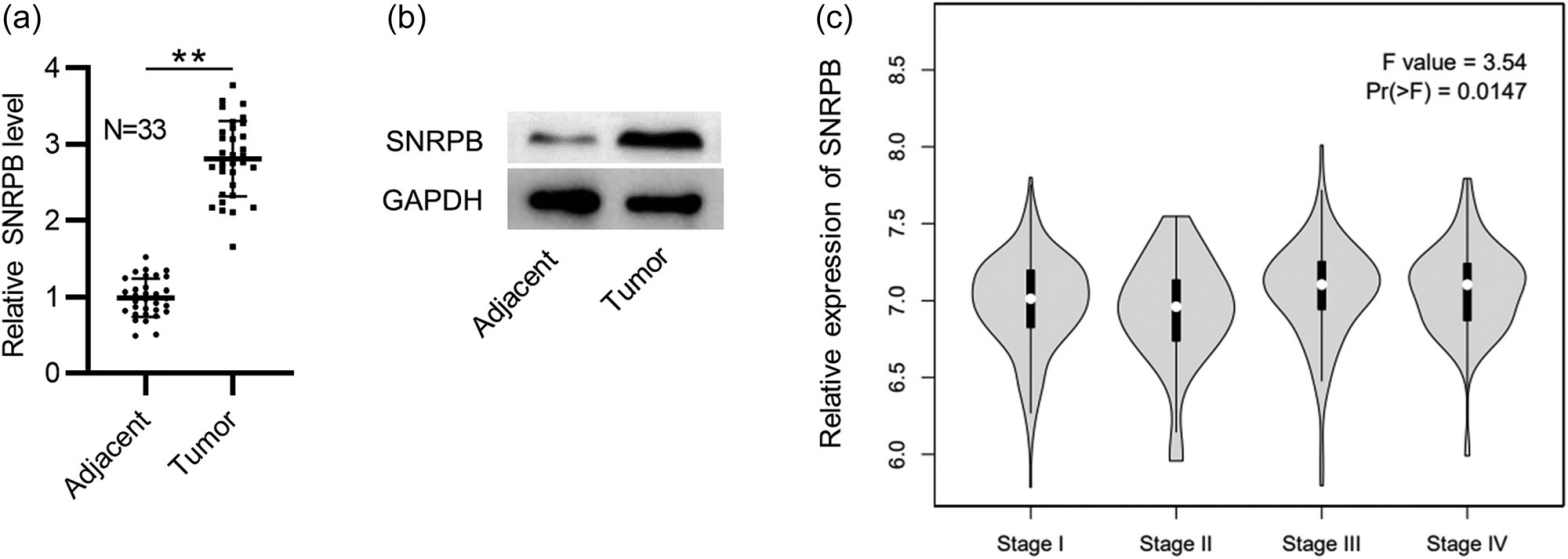
SNRPB displays a high level in PTC: (a and b) RT-qPCR analysis and western blotting of SNRPB mRNA and protein levels in PTC and adjacent normal tissues (*N* = 33) and (c) SNRPB expression in THCA tissues of different stages shown by GEPIA. Student’s *t-*test was performed for statistical analysis. **p < 0.01.

First, we tested SNRPB expression in PTC tissues by RT-qPCR together with western blotting. In comparison to those in the adjacent nontumor samples, mRNA and protein levels of SNRPB were elevated in PTC tissues (*N* = 33) (Figure 1a and b). Moreover, data from GEPIA revealed that SNRPB expression is higher in PTC tissues at stage III and IV than those at stage I and II (Figure 1c), indicating the close relation between SNRPB level and PTC development.

### 3.2 SNRPB silencing suppresses PTC cell cycle progression

**Figure 2:**
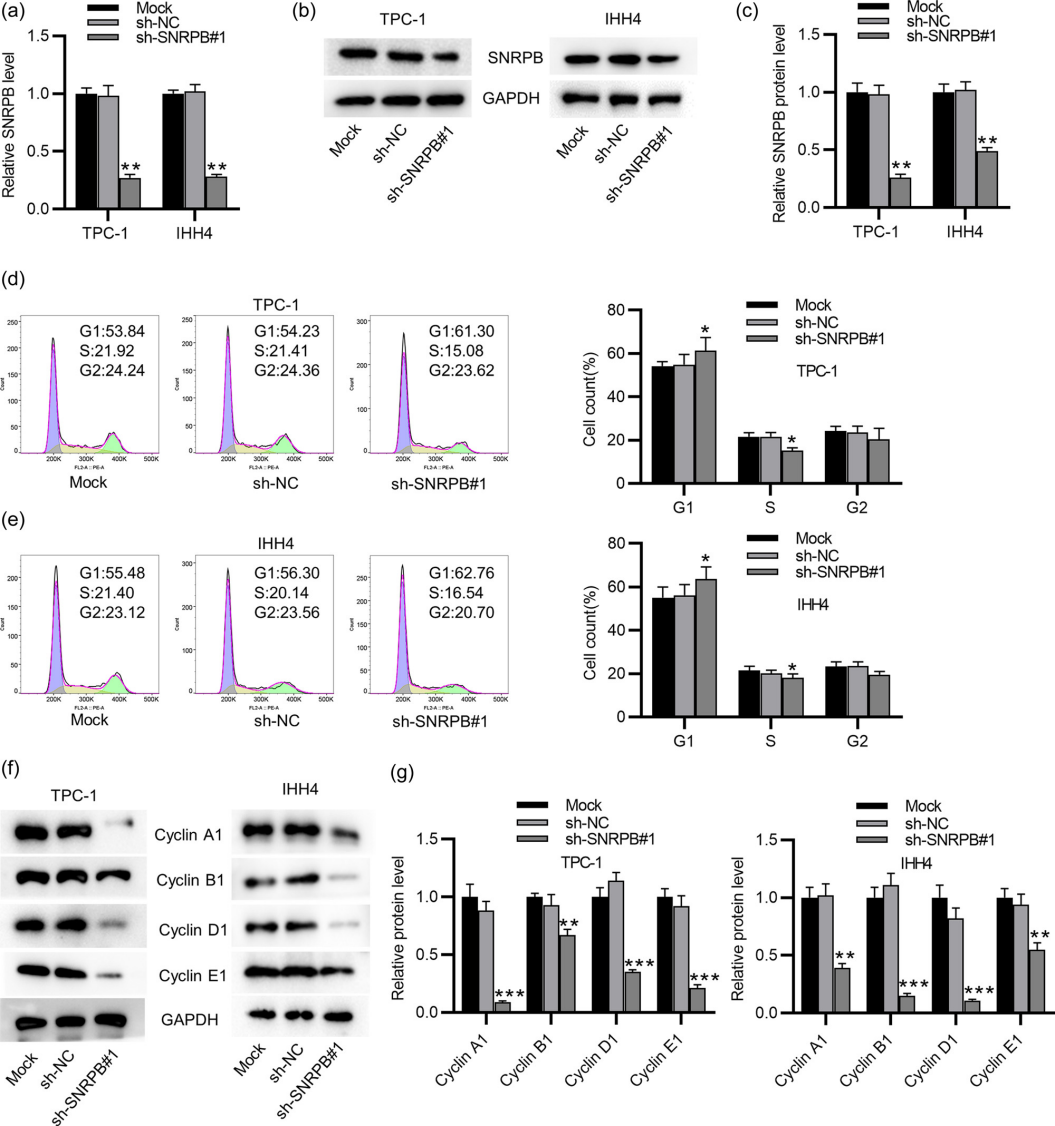
SNRPB knockdown induces G1 phase arrest in PTC cells: (a–c) RT-qPCR and western blotting for evaluating efficiency of SNRPB knockdown in PTC cells (TPC-1, IHH4), (d and e) flow cytometry for determining cell cycle distribution of PTC cells with treatment of mock, sh-NC or sh-SNRPB#1 and (f and g) western blotting for assessing levels of cell cycle-associated proteins in indicated PTC cells. ANOVA followed by Tukey’s *post hoc* analysis was performed for statistical analysis. *p < 0.05, **p < 0.01, ***p < 0.001.

To test SNRPB effect on malignant phenotypes of PTC cells, we knocked down SNRPB in two PTC cell lines (TPC-1 and IHH4). The efficiency of SNRPB was confirmed by RT-qPCR and western blotting which showed that SNRPB levels were markedly reduced in sh-SNRPB#1-transfected cells (Figure 2a–c). Subsequently, SNRPB effect on cell cycle progression was evaluated by flow cytometry. It was shown that the percentage of cells in G1 phase was dramatically enhanced in sh-SNRPB#1-treated group, compared to the mock- and sh-NC-treated groups (Figure 2d and e). Additionally, SNRPB silencing presented a significant reduction in the percentage of cells in S phase (Figure 2d and e). These results suggested that knocking down SNRPB induced cell cycle arrest in G1 phase. This was confirmed by the results of western blotting in detection of cell cycle-related proteins. As shown in Figure 2f and g, protein levels of Cyclin family (A1, B1, D1, E1) were decreased in sh-SNRPB#1-transfected PTC cells. Overall, depletion of SNRPB restrains PTC cell cycle progression.

### 3.3 SNRPB expression is positively correlated with cell cycle-related gene expression

**Figure 3:**
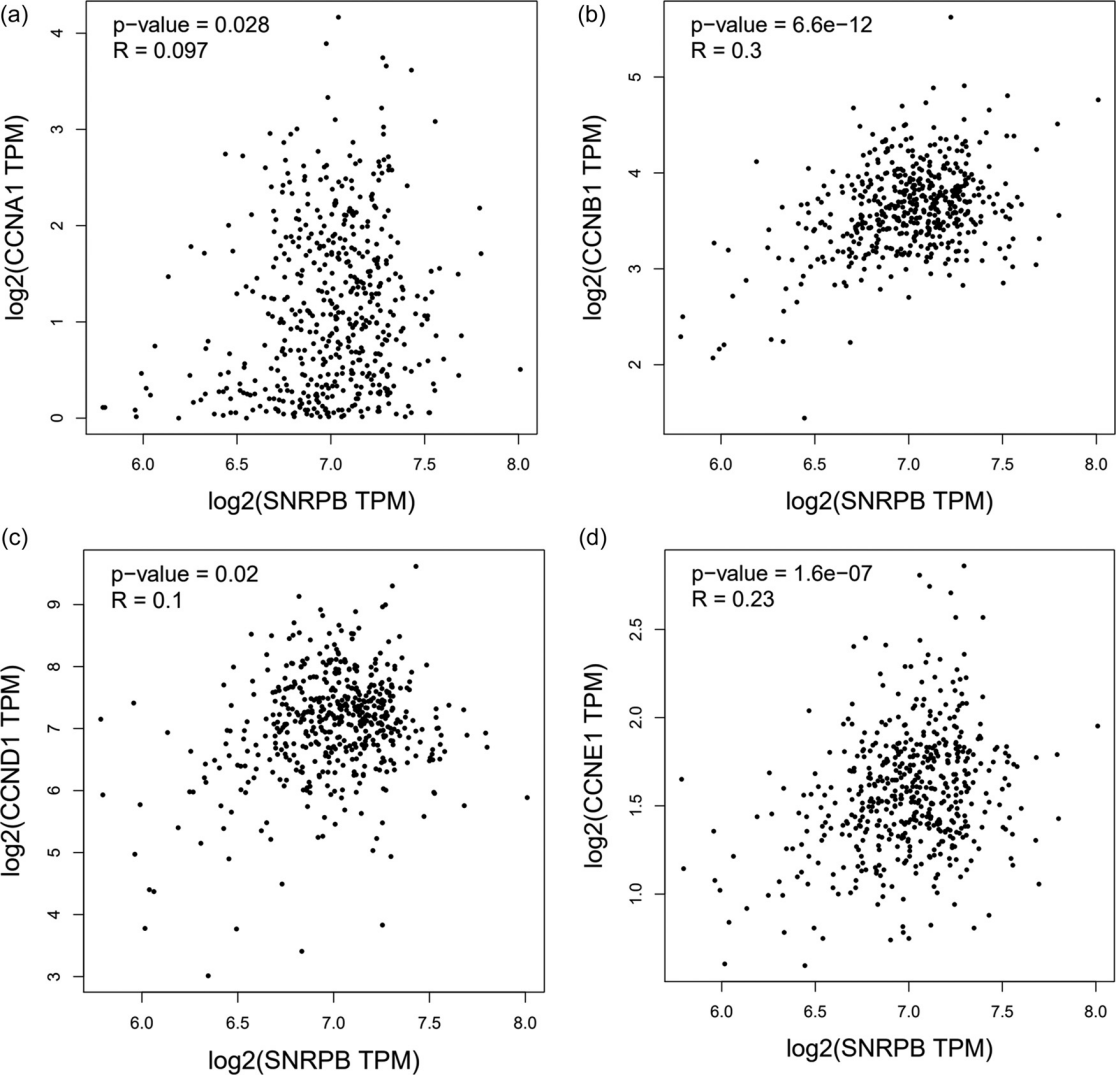
SNRPB expression is positively correlated with cell cycle-related gene expression: (a–d) expression correlation between SNRPB and cell cycle-associated genes in THCA tissues shown by GEPIA database.

Cyclin family proteins are encoded by corresponding cell cycle-related genes (CCNA1, CCNB1, CCND1 and CCNE1) [19]. Notably, data from GEPIA database displayed that SNRPB expression has a positive correlation with cell cycle-related gene expression in THCA tissues (Figure 3a–d), further confirming that SNRPB is strongly associated with the development of THCA.

### 3.4 SNRPB inhibits p53 expression

**Figure 4:**
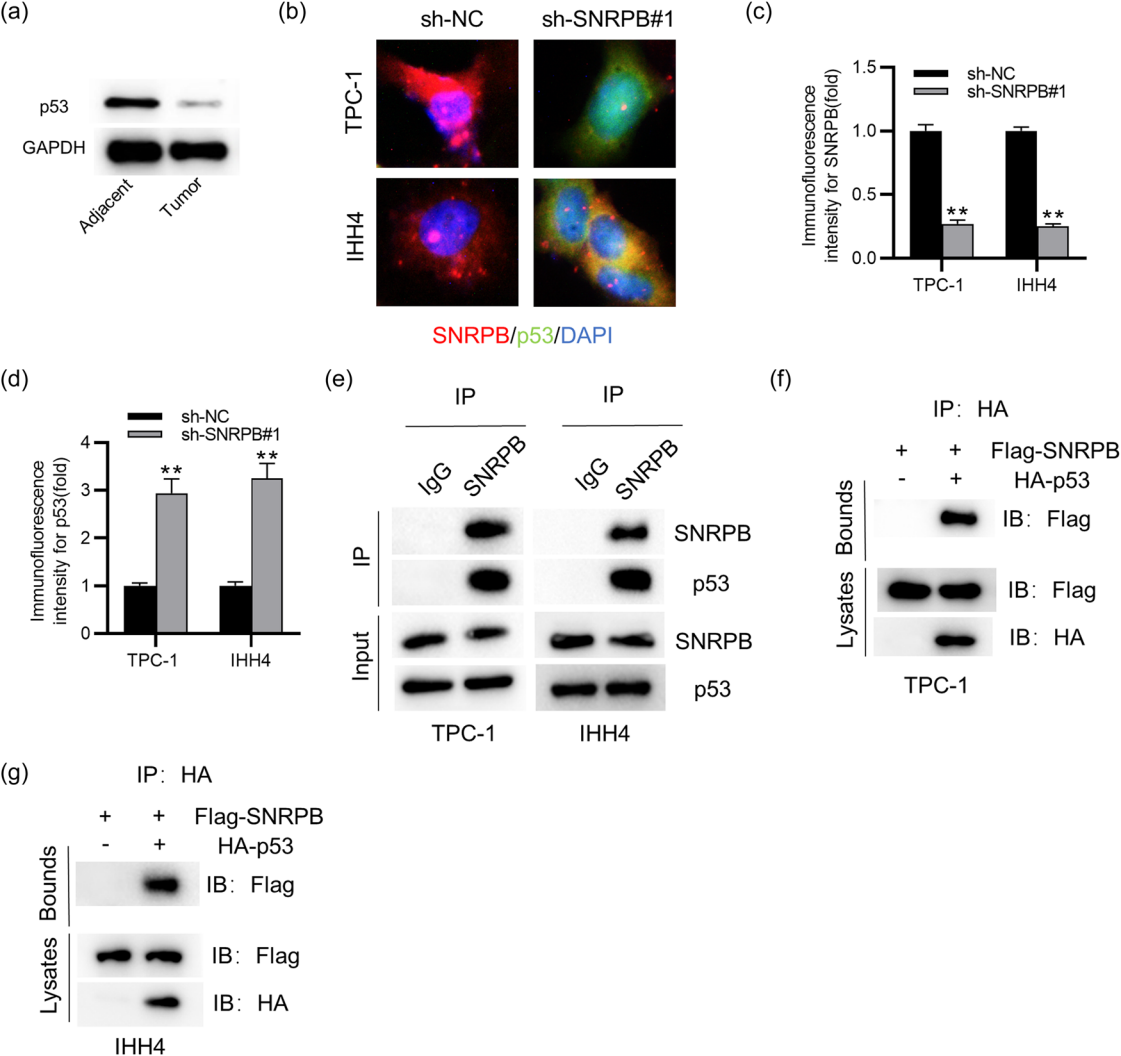
SNRPB inhibits p53 expression: (a) western blotting of p53 protein level in PTC tissues and adjacent normal tissues, (b) IF staining for detecting expression of SNRPB and p53 in sh-NC- or sh-SNRPB#1-transfected PTC cells, (c and d) quantification of IF intensity of SNRPB and p53 and (e–g) Co-IP for determining the interaction between p53 and SNRPB in PTC cells. Student’s *t-*test was performed for statistical analysis. **p < 0.01.

It has been indicated that SNRPB exerts an oncogenic role in cervical cancer by inhibiting p53 expression [17]. Here, to reveal the underlying mechanism of SNRPB in PTC, we detected its impact on p53 in PTC cells. We first detected the protein level of p53 in PTC tissues. The results of western blotting displayed that p53 protein expression was downregulated in PTC tissues in comparison to that in the adjacent normal samples (Figure 4a). As shown by IF staining, SNRPB was markedly expressed in PTC cells compared with that of p53 (Figure 4b). Notably, knocking down SNRPB led to a significant increase of p53 expression in PTC cells (Figure 4b–d). Furthermore, the results from Co-IP presented that SNRPB could interact with p53 in PTC cells (Figure 4e–g). To improve the specificity of detection, SNRPB and p53 were fused with protein tags to generate Flag-SNRPB and HA-p53. It was confirmed that SNRPB directly interacted with p53 in PTC cells (Figure 4f and g). Collectively, SNRPB directly interacts with p53 and inhibits its expression in PTC cells.

### 3.5 Knocking down SNRPB restrains tumor growth *in vivo*

**Figure 5:**
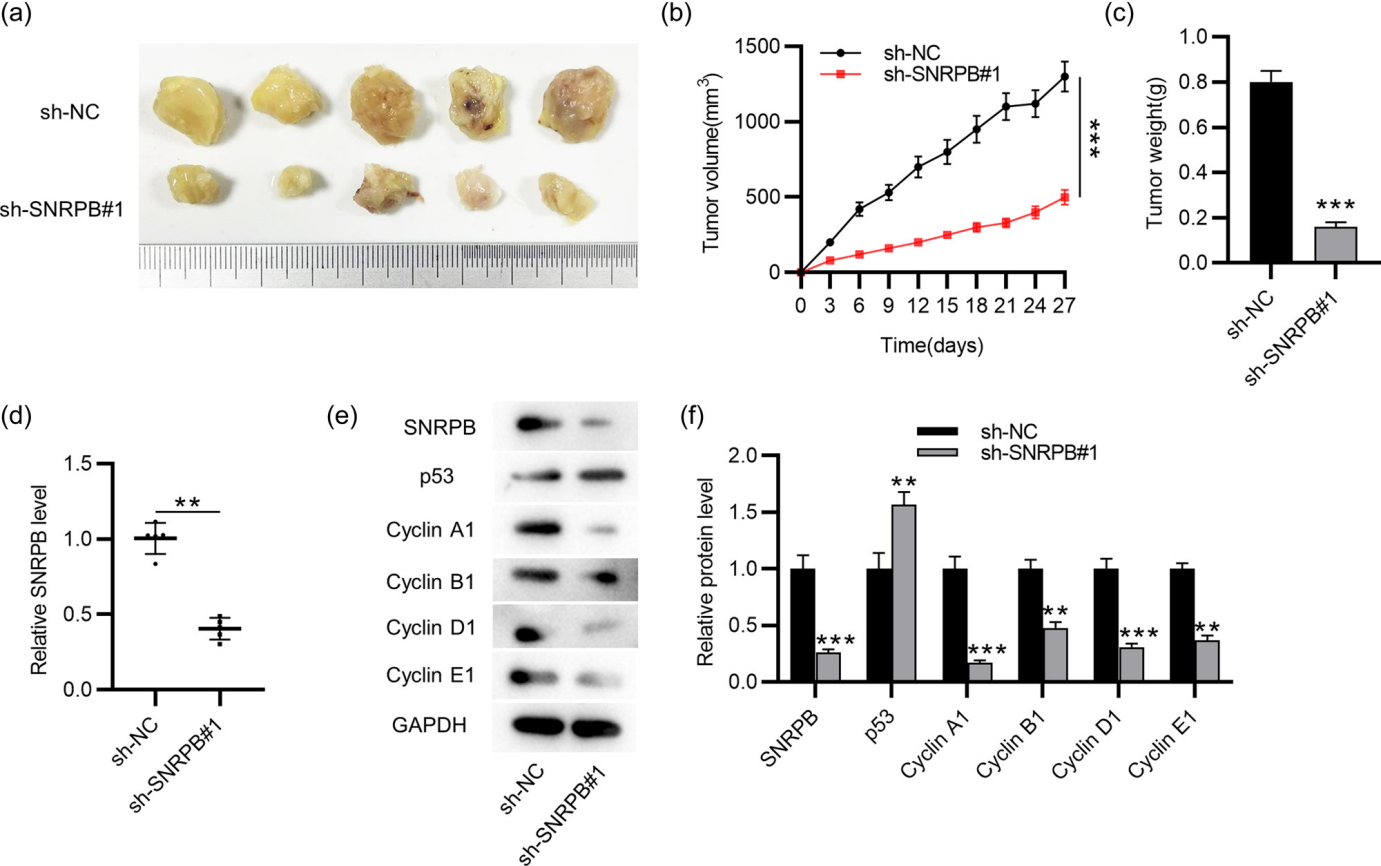
Knocking down SNRPB restrains tumor growth *in vivo*: (a) photographs of murine tumor samples, *n* = 5 per group, (b) tumor volumes of each group measured every 3 days, (c) measurement of tumor weight on the 27th day, (d) RT-qPCR analysis of SNRPB level in each group and (e and f) western blotting for evaluating levels of SNRPB, p53 and cell cycle-related proteins in murine tumors. Student’s *t-*test was performed for statistical analysis. **p < 0.01, ***p < 0.001.

To further elucidate the function of SNRPB in PTC, *in vivo* experiments were implemented. Mice were injected subcutaneously with sh-NC- or sh-SNRPB#1-transfected TPC-1 cells. The results displayed that the tumors were smaller and grew more slowly in SNRPB-depleted group (Figure 5a–c), indicating that knocking down SNRPB might suppress PTC tumor growth. Additionally, compared to that in sh-NC-treated group, SNRPB level in sh-SNRPB#1-treated group was markedly decreased (Figure 5d). Moreover, SNRPB protein level was reduced in SNRPB-depleted group, while that of p53 was significantly elevated (Figure 5e and f). Notably, depletion of SNRPB resulted in the downregulation of cell cycle-associated proteins in tumors (Figure 5e and f), validating that SNRPB silencing inhibits cell cycle progression *in vivo*.

## 4 Discussion

PTC is the major type of THCA which accounts for over 80% of all THCA cases, with the majority of cases in women [20]. Most of PTC cases are associated with favorable prognosis; however, in certain cases, patients tend to develop clinical aggressive disease and have a high rate of recurrence after treatments [21]. Hence, it is imperative to understand the mechanism underlying PTC pathogenesis and find novel effective approaches for PTC treatment.

Numerous studies have revealed the critical role of SNRPB, a core component of spliceosome, in tumorigenesis, including cervical cancer, glioblastoma and non-small cell lung cancer [7,9,17]. SNRPB exerts a carcinogenic role in the progression of many cancers by promoting malignant behaviors of cancer cells. For example, SNRPB overexpression facilitates the proliferative and migratory capabilities of hepatocellular carcinoma cells [22]. In cervical cancer, SNRPB upregulation enhances cell proliferation but inhibits cell apoptosis [17]. Additionally, dysregulation of SNRPB is closely associated with the adverse prognosis of patients [22]. To our knowledge, it is unclarified whether SNRPB is involved in PTC progression. In accord with previous evidence, the results from this study revealed the overexpression of SNRPB in PTC tissues. Additionally, bioinformatics analysis presented that SNRPB is differentially expressed in different stages of THCA patients, with a higher level in stage III/IV. These indicated that high expression of SNRPB has a strong correlation with the aggravation of PTC. To validate this, loss-of-function assays were implemented to examine the detailed function of SNRPB in PTC. It was shown that depleted SNRPB markedly restrained cell cycle progression of PTC cells. Additionally, a positive correlation between SNRPB expression and cell cycle-associated gene expression in THCA tumors is presented by data from GEPIA database, further confirming SNRPB effect on PTC cell cycle. Consistently, *in vivo* experiments demonstrated that SNRPB silencing suppressed tumor growth in mice. Overall, these results revealed that SNRPB can promote PTC progression *in vitro* as well as *in vivo*.

Tumor suppressor p53 is activated in response to various cellular stresses including oncogenic signaling and works as a transcription factor to modulate target gene expression, consequently inducing cell cycle arrest, DNA repair, apoptosis or metabolic changes [23,24]. p53 is considered as one of the most vital genes in protecting against human cancers [25]. Importantly, a previous study demonstrated that p53 expression is inhibited by SNRPB in cervical cancer [17]. Here, to probe the potential mechanism of SNRPB in PTC, we tested its effect on p53 expression in PTC cells. The results displayed that SNRPB directly interacted with p53 and knocking down SNRPB enhanced p53 expression, indicating that SNRPB suppressed p53 expression in PTC cells. Furthermore, it has been reported that the Wnt/β-catenin pathway is a major target of p53, and the β-catenin pathway promotes the proliferation and metastasis of PTC cells [26,27]. This indicates that the Wnt/β-catenin signaling pathway might be involved in SNRPB/p53-mediated PTC, which needs to be further investigated in the future.

In conclusion, we probed the function of SNRPB as well as its underlying mechanism in PTC. The results displayed that SNRPB is highly expressed in PTC tissues and SNRPB silencing restrains cell cycle progression of PTC cells and inhibits tumor growth in mice largely by suppressing p53 expression. The findings might develop a novel biomarker for PTC diagnosis and a new clue for PTC treatment. Additionally, the potential role and mechanism mediated by SNRPB/p53 in PTC need further investigation in the future.
